# The Association between Operating Room Nurses' Characteristics, Competence, and Missed Nursing Care: A National Survey

**DOI:** 10.1155/2023/6671866

**Published:** 2023-12-08

**Authors:** Brigid M. Gillespie, Emma Harbeck, Wendy Chaboyer

**Affiliations:** ^1^National Health and Medical Research Council Centre of Research Excellence in Wiser Wound Care, Menzies Health Institute Queensland, Griffith University, Gold Coast 4222, Australia; ^2^Gold Coast University Hospital, Gold Coast Health Nursing and Midwifery Education and Research Unit, Queensland, Gold Coast 4215, Australia

## Abstract

**Background:**

Missed nursing care, which has been explored in various acute care settings, results in adverse patient outcomes and job dissatisfaction in nurses. However, little is known about missed care in the operating room.

**Objective:**

This study tested a hypothesised model to identify relationships between nurses' age, years of experience in the operating room, job satisfaction, and intention to leave which have direct and indirect effects on the frequency of missed care. The frequency of missed care was hypothesised to be mediated by nurses reported perioperative competence and the reasons for missed care.

**Design:**

A cross-sectional design using an online survey of Australian perioperative nurses was undertaken in 2022.

**Methods:**

All Australian College of Perioperative Nurses members were invited to participate. Missed nursing care was measured using the MISSCARE Survey-OR. Age, years of experience, and intention to leave were single-item measures. Satisfaction was a three-item scale. Competence was measured by the 18-item Perceived Perioperative Competence Scale-Short Form. Structural equation modelling was used to test our hypothesised model.

**Results:**

Of the 5,500 nurses invited, 853 (15.5%) responded, but only 602 (10.9%) participant responses were usable for inclusion in the model. The model demonstrates that participants' age directly predicted the frequency of missed care, nurse role satisfaction, perceived perioperative competence, and reasons for missed care. The reasons for missed care and perceived perioperative competence were mediators that were negatively associated with the frequency of missed care.

**Conclusions:**

While the final model explained 22.6% of the frequency of missed care, other variables not identified in this study may influence this outcome.

## 1. Introduction

Missed care is any aspect of care that is omitted or delayed, in part or whole [1]. There is a large body of research on missed nursing care in inpatient units, including medical-surgical and intensive care units [2–5]. One review of 18 studies identified that 75% or more of nurses reported omitting care [6]. An overview of seven reviews identified missed care fell into four categories: communication and information sharing; self-management, autonomy, and education, including care planning, discharge planning, and decision; fundamental physical care; and emotional and psychological care, including spiritual support [7]. An integrative review of 54 papers identified that a combination of nurse, patient, and organizational contextual variables explained 12 to 32% of the variance in missed care across studies reporting this [3]. This same review acknowledged that predictors of missed nursing care included perceived team interactions, adequacy of resources, safety climate, and nurse staffing. Patient outcomes negatively affected by missed nursing care include hospital-acquired infections, pressure injuries, falls, discharge planning, mortality, patient mobilisation, feeding, and psychological and emotional support [1, 8–11]. Clearly, missed care may compromise patient safety [7].

Several systematic reviews [3, 4, 6, 12–14] and an overview of reviews [7] have identified predictors of missed nursing care across a variety of hospital contexts but predominantly in medical-surgical settings [7]. Across these reviews, factors found to be associated with missed nursing care included skill mix, i.e., combination and distribution of clinical skills and competencies in the nursing workforce [6], staffing levels [3, 6, 13], a lack of material resources [3], patient acuity [3, 13], and teamwork and communication [3, 12, 13]. Only one study of missed nursing care in the operating room (OR) setting was identified. In a survey of 1,593 US OR nurses, Marsh et al. [15] found significant associations between the number of operating rooms at a facility, nurse education, job title, perceptions of staffing adequacy, and frequency of missed perioperative nursing care.

Kalisch's Missed Nursing Care Model [1, 11] has been seminal in this field. The Missed Nursing Care Model illustrates the various attribute categories nurses report in acute care settings that contribute to missed nursing care. This framework examines three concepts; structure (e.g., hospital, patient care unit, and nurse characteristics), process (missed care), and outcomes (staff outcomes such as job satisfaction and patient outcomes). Interestingly, despite the importance of nurse characteristics in this model, there is a paucity of research on the effect of nurses' competence on missed nursing care.

Competence broadly encompasses nurses' knowledge, skills, and attitudes required to undertake their professional role and clinical practice effectively and safely [16]. While these generic attributes apply across any nursing setting and speciality, they do not reflect the behavioural markers that specifically apply to specialities such as OR nursing [17]. In this highly specialised context, competence is conceived relative to technical and nontechnical skills [18, 19]. For instance, technical skills encompass practical and foundational knowledge, whereas nontechnical skills include teamwork, communication, leadership, and holistic and empathic care [19–21]. Competence is a critical determinant of role performance, and thus, it is reasonable to assume that perioperative competence is related to the quality of care that nurses provide. However, paradoxically, the relationship between perioperative competence and missed nursing care has never been tested.

### 1.1. Missed Nursing Care in OR Nursing: A Hypothesised Model

**Figure 1:**
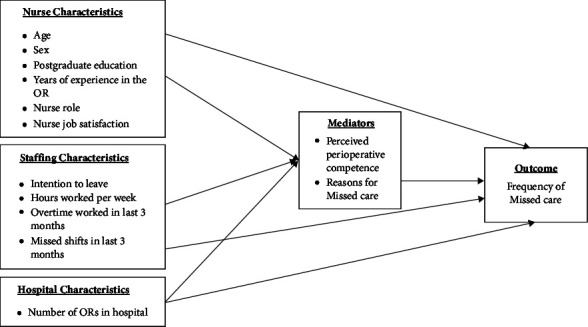
Hypothesised model indicating expected pathways of association.

We have built on Kalish's model to incorporate nurse competence as an essential structural predictor of missed nursing care (Figure 1). The empirical relationships between nurses' demographic characteristics, unit, and missed care have been well established across a body of research over the past two decades [2, 8, 9, 22–24]. The results of several studies also suggest that nurse job satisfaction and staffing levels contribute to missed care [2, 11, 13, 22, 24]. Kalisch and others examined the relationship between missed care and staff outcomes of job satisfaction, intention to leave, hours worked per week, and overtime as outcomes. However, in our hypothesised model, we have treated these staff outcomes as potential predictors of the frequency of missed care. Therefore, in this model, hours worked and overtime have been considered surrogates of staff fatigue, which may contribute to missed care. We also hypothesised that there is a relationship between the number of ORs and the frequency of missed care: larger OR departments have implications for an increased workload and time pressures, staffing, and resources. Additionally, the relationship between reasons for missed care and its frequency has not previously been established. We hypothesised that nurses' age, years of OR experience, job satisfaction, intention to leave, hours and overtime worked, and the number of ORs and reasons for missed care have direct effects on the reported frequency of missed care.

However, we also wanted to describe the contribution of nurses' perceived perioperative competence on the reported frequency of missed care. Safe nursing care in the OR environment requires competence in practice, as nurses need to apply nontechnical skills such as coordination, communication, teamwork, and empathy [19]. Therefore, we hypothesised that nurses perceived perioperative competence would be inversely related to the frequency of missed nursing care and mediate the effect of nurse, staff, and hospital characteristics. Findings generated through this study may help nurse managers identify areas of OR practice where missed nursing care most often occurs and enable them to develop strategies that specifically target these areas.

## 2. Materials and Methods

### 2.1. Design, Setting, and Sample

A cross sectional design using an online survey design was used. Two members of the research team pilot tested the survey for flow, structure, and ease of navigating the electronic interface. A census of all 5,500 operating room nurses who were members or associate members of the Australian College of Perioperative Room Nurses (ACORN) was undertaken in 2022. Eligible participants included registered nurses (RNs) working in clinical (i.e., circulating, instrument, anaesthetic, and recovery room roles) and education roles across public and private sectors. Enrolled nurses were excluded due to the differences in their scope of practice. The conduct and reporting of the study were guided by the Strengthening the Reporting of Observational Studies in Epidemiology (STROBE) Statement [25].

### 2.2. Measures

The online survey had three components measuring (1) demographic characteristics; (2) preoperative and intraoperative missed care; and (3) perceived perioperative competence. The survey had 111 items and took approximately 25 minutes to complete. Fifteen questions were used to collect data on participants' age, sex, state or territory of residence, clinical role, years of OR experience, highest qualifications, job satisfaction, hours of overtime worked over the past three months, intention to leave current position, and staffing levels. Job satisfaction was measured using a 5-point Likert scale to indicate the degree of satisfaction ranging from *very satisfied* to *very dissatisfied* [24, 26]. We recoded this variable so higher scores indicated higher job satisfaction.

#### 2.2.1. The MISSCARE Survey-Operating Room

Part A includes elements of preoperative and intraoperative missed nursing care, and part B includes reasons for this missed care [15, 27]. The 53-item survey with five subscales has been psychometrically validated in a US sample of 1,693 operating room nurses and has five subscales: legal, preparation, safety, communication, and closing routine. Response options for the preoperative and intraoperative care items include *never, rarely missed, occasionally missed, always,* and *not applicable*. Response options for reasons for missed care include *significant, moderate, minor,* and *not a reason for missed care*. This measure was also recoded for interpretation, where higher scores indicated the item was frequently perceived as missed in the preoperative and intraoperative periods, as well as higher scores indicating agreement that the reason presented was a significant reason care was missed.

#### 2.2.2. Perceived Perioperative Competence

Competence was measured with the Perceived Perioperative Competence Scale-Short Form (PPCS-SF) [28]. The scale has six dimensions of perioperative competence: foundational knowledge, proficiency, professional development, leadership, collaboration, and empathy. The short form 18-item scale was derived and psychometrically validated using items from the previously validated 40-item PPCS-revised form [19]. The PPCS-SF has been validated and includes the same six subscales that indicate different dimensions of perioperative competence: foundational knowledge, proficiency, professional development, leadership, collaboration, and empathy. Total scores for each subscale can be calculated and considered separately. Both measures were developed in Australia and tested with over 1,500 operating room nurses. Like the previous revised version, the short form has a five-point Likert scale: *never (1), sometimes (2), often (3), very often (4),* and *always (5)*. Scale scores range from 18 to 90, with higher scores indicating greater levels of perioperative competence.

### 2.3. Data Collection

The university's research data capture (REDCap) hosted the online survey [29, 30] data management system. Recruitment was active for six weeks (March–April 2022), with three reminders emailed fortnightly to potential participants.

### 2.4. Data Analyses

Survey data were exported into SPSS version 27 (Armonk, NY: IBM Corp.) for data cleaning and assumption checks. The types of descriptive analyses used to describe the sample and both the competence and MNC scales were determined by the level of the data (i.e., categorical or continuous) and its distribution. For example, frequencies, including numbers and percentages, were used for categorical variables (e.g., gender, role, and type of facility), while means and standard deviation were calculated for continuous variables (e.g., age, years of OR experience, and scales: MISSCARE-OR and PPCS-SF).

In inferential analyses, the dependent variable was the overall missed nursing care score, based on the average amount of reported missed nursing care identified for each of the elements of nursing care for each participant on a five-point scale (i.e., 0 = *never missed* to 4 = *always missed*). Based on our hypothesis, we used a model-building approach [31], starting with bivariate correlations to determine associations between demographic factors (i.e., age, years of OR experience, education qualifications, role, and hospital facility), nurse factors (i.e., perceived perioperative competence, overtime worked, job satisfaction, intention to leave, and staffing levels), and missed nursing care. Factors that shared a direct relationship that were statistically significant (*p* < 0.05) were subsequently entered into an SEM to predict any direct or indirect effects on the frequency of missed care in the operating room.

SEM was used because it allowed for the testing of both direct (observed) and indirect (latent) effects while accounting for mediating variables [32]. Additionally, one of the greatest advantages of using SEM is its ability to manage measurement error which may potentially weaken parameter estimates. The final hypothesised SEM was fitted to a covariance-based structural equation model with a maximum likelihood estimator using the lavaan package in R (lavaan version 0.6-13, R version 4.2.2. RStudio version 12.0). Standard errors and associated *p* values were calculated using the robust maximum likelihood (mlr) to account for any non-normality in the data. Maximum likelihood (ml) was also applied to account for missing data. Model fit was assessed using *χ*^2^ Goodness-of-Fix Index, the Comparative Fit Index (CFI), and the Tucker–Lewis Index (TLI), which all should be above 0.9 for acceptable fit; the standardised root mean squared residual (SRMR), which should be below 0.08; and the root mean square error of approximation (RMSEA), which also should be below 0.08 and not significantly different from 0.05 (*α* = 0.05, one-tailed). Standardised factor loadings over 0.4 were considered acceptable [33].

### 2.5. Ethical Considerations

Submission of the survey in the REDCap system implied consent. The study was reviewed and approved by the author's Human Ethics Review Board (HREC Griffith University reference number 2021/774).

## 3. Results

### 3.1. Sample Characteristics

Sample characteristics are displayed in Table 1. The total number of respondents was 853 (15.5% of an estimated 5500 invited). However, of the 853 that engaged with the survey, only 602 (70.6%) provided data that could be analysed as not all participants completed the demographic items. Thus, the sample used in the SEM was 602. The average age of the sample was 46 years (SD 11.4). Respondents were predominately female (82.6%), and their primary OR role was circulating/instrument nurse (50.5%). Under half of the sample held a bachelor's degree in nursing (40.4%). However, 121 (20.1%) reported having postgraduate education. The sample was highly experienced operating room nurses, with 64.1% indicating they had worked in an operating room for 10 or more years. All surgery types and surgical specialists were represented in this sample.

### 3.2. Model Building

Before testing the model, a correlation matrix was used to examine the initial predictor relationships with the frequency of missed nursing care (Table 2). All potential predictors with a significant direct relationship were entered into the SEM model. The correlation matrix showed that the following variables were statistically associated with the reported frequency of missed care in the operating room: reasons for missed care in the operating room, perceived perioperative competence, age (years), intention to leave, and nurse satisfaction. Additionally, years of experience in the operating room were added to the model due to its moderate association with perceived OR experience. Overall, Spearman's correlations were weak to moderate (|*r*| = 0.03–0.37).

### 3.3. SEM of Frequency of Missed Care in the Operating Room

**Figure 2:**
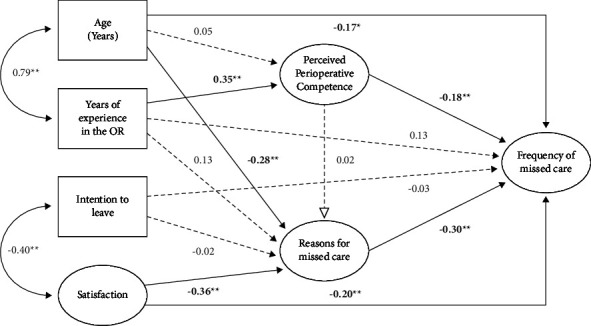
Final model with standardised coefficients. *Note*. The dashed lines with arrows represent nonsignificant relationships (*p* > 0.05). ^*∗*^*p* < 0.05, ^*∗∗*^*p* < 0.01. The dashed lines with arrows represent nonstatistically significant relationships (*p* > 0.05). ^*∗*^*p* < 0.05, ^*∗∗*^*p* < 0.01.

The model testing results demonstrate that participant age predicted the frequency of missed care, whereas younger participants reported higher frequency and satisfaction. In contrast, those with lower satisfaction in nursing roles reported a higher frequency of missed care. In relation to perceived perioperative competence, those with higher perceived competence reported less frequency and reasons for missed care. Respondents who reported higher agreement for potential missed care reasons also reported a higher frequency of missed care observed in their workplace. Reasons for missed care was the strongest predictor *β* = 0.30, followed by satisfaction *β* = −0.20 and perceived perioperative competence *β* = −0.18. However, in contrast to our hypothesised model, intention to leave in the next six months and years of OR experience in the operating room did not directly predict the reported frequency of missed care. Age and satisfaction also indirectly affected the frequency of missed care via reasons for missed care, as well as years of experience in the operating room mediated through perceived perioperative competence (Table 3). The total effect of age on the frequency of missed care was −0.23, while satisfaction was −0.32 and years of experience in the operating room was −0.10 (Table 3). The final model is displayed in Figure 2. The model predicted 15.1% of the variance in perceived perioperative competence, 15.9% of the variance in reasons for missed care, and 22.6% of the variance in frequency of missed care. All parameter estimates, including indirect and total effects, are presented in Table 3.

The analysis indicated an acceptable model fit to the data on most indices (CFI = 0.90; TLI = 0.90; RMSEA = 0.04 (0.037, 0.040), *p* =1.00; and SRMR = 0.051) (Figure 2). However, the Chi-square value did not support good model fit (*χ*^2^ (2499) = 4733.45, *p* < 0.001). In the measurement models, all items were significantly loaded onto their respective factors (all *p*'s <0.05), and no items had negative variances or negligible standardised loadings (all loadings >0.4).

## 4. Discussion

To our knowledge, this is the first study to identify factors predictive of reported missed OR care using SEM. Previous research examining missed OR care [15] has only investigated the types and frequencies and relationships of missed care to facility characteristics. Generally, our findings relative to some nurse characteristics support the broader research in this area. Twelve nurse and hospital characteristics predictors were entered into the initial model as part of a model-building approach, but only five were explored in the final SEM. Moreover, some model paths were not statistically significant (i.e., intention to leave in the next six months and years of OR experience). We found that nurse age and job satisfaction had direct and indirect effects, and years of experience only indirectly influenced participants who reported perceived missed care in the OR. Reasons for missed care and perceived perioperative competence were mediators in the model. Relationships between reasons for missed care, perceived perioperative competence, and missed care have not been tested in *any* nursing context and thus are novel, adding new knowledge to what is already known. Notably, the reasons for missed care and perceived perioperative competence are amenable to change via interventions. Therefore, these results identify areas that nurse managers can focus on when developing strategies to address the reasons for missed care.

### 4.1. Mediators of Missed Perioperative Nursing Care

Reasons for missed perioperative care were the strongest predictor contributing to the reported frequency of missed OR care. Reasons for missed nursing care include team communications and interruptions, limited resources, and staff factors such as inadequate skill mix and staffing. Inadequate skill mix in the OR environment has been formerly identified as a major patient safety issue in previous research [34, 35]. An earlier literature review of 14 studies identified that lower staffing levels contributed to missed care across various nursing environments [6, 24]. In our study, other items with high loadings on the Reasons for Missed Care scale related to teamwork and communication breakdowns and lack of support from coworkers. Patients are at their most vulnerable during surgery; as such, the importance of effective teamwork and communication for safe OR care cannot be overstated [18, 19, 36, 37]. Notably, there is an even greater imperative for team members to work cohesively together in this high-dependency context [35–37]. Plausibly, ineffective teamwork in the OR can result in communication breakdowns, reduce efficiency and coordination, increase errors, and create an environment lacking support. Ultimately, this leads to job dissatisfaction, which in our study has been identified as a predictor of the frequency of missed nursing care. There is an abundance of research undertaken in OR contexts [36–39] describing the barriers to effective teamwork and communication among members of the interdisciplinary team. Our results support previous research undertaken in med-surg environments [11, 40] that has identified a lack of teamwork as a predictor of missed nursing care. Clearly, the reasons for missed care are highly correlated to missed care and can potentially compromise patient safety.

Importantly, this study found a direct relationship between perceived perioperative competence and the frequency of missed care. This is the first study to identify a direct relationship between these constructs. Arguably, it is reasonable to expect that nurses reporting higher levels of perceived perioperative competence would also report fewer occasions of missed care. Competent nurses are often better at managing their time effectively, which can help them prioritise their tasks and ensure that important nursing care is not missed [16, 40, 41]. Additionally, more competent nurses are likely to have better clinical judgment. This can help them identify tasks requiring priority or immediate attention and ensure appropriate nursing care is provided [16, 19]. Second, competent nurses are likely to have a higher level of knowledge and skills related to patient care, which can help them provide more comprehensive and effective nursing care [17]. Third, competent nurses may be more confident and assertive in their interactions with patients and other healthcare professionals. This can help them advocate for their patients and ensure that important nursing care (e.g., pressure area care and intraoperative warming) is not overlooked.

The pressure to complete operations within tightly scheduled timeframes and finite resources may contribute to missed care. Additionally, there is often a disconnection between the priorities of surgeons, anaesthetists, and team nurse members [38]. Thus, establishing a hierarchy of priorities is important but not easy in this environment, as nurses must work interdependently with other disciplines, and these members have a different focus. OR nurses need to differentiate between care that must be immediately provided and care that can wait. Perhaps some tasks are viewed by nurses as not having any intermediate or measurable impact on patient outcomes, e.g., pressure area care, providing comfort measures, and communication of essential information. The results of this study may have implications for OR nurse education and on-the-job training. The emphasis on particular nursing tasks in the OR may shape the novice nurse's attitudes at the beginning and during their professional transition in deciding how to prioritise which care activities can be missed.

### 4.2. Limitations

We acknowledge several study limitations. First, because it was cross sectional, we identified associations but could not conclude causation; that is, our hypothesised predictors were the cause of the frequency of missed nursing care. Thus, results need to be interpreted with caution. Second, we cannot rule out selection bias despite inviting all OR nurses in Australia's peak professional organisation. Moreover, of the 5,500 perioperative nurse members, only 602 responded. To mitigate nonresponse bias, the survey was available for six weeks, with two email reminders and social media posts used to help advertise the survey (e.g., the ACORN Facebook page). Though the sample of those who responded was small, it was represented for all Australian states, and surgical specialities. Third, variable effects were weak to moderate, and the total amount of variance explained by the model was 23%. This low amount of variance explained by our model indicates that other variables that influence the reported frequency of missed care in the operating room have not been identified.

Furthermore, while model fit statistics were acceptable, modification indices were not explored and may suggest measurement model improvements could be made. However, modification indices represent a statistical decision approach, and our model relied on previously validated scales (e.g., MISSCARE-OR, job satisfaction, and PPCR-SF). Thus, model improvement was not undertaken by removing poorly performing items, or correlating item and or latent variable pathways that were not hypothesised. Finally, this study's data source may be biased because all study variables were nurse reported. Future studies could include source data from patients to confirm the results, even though nurse-reported patient safety indicators have been used widely in research and are proven to be substantially associated with patient outcomes.

### 4.3. Implications for Nursing Management

The results of our study suggest that younger, more job-dissatisfied nurses reported higher frequencies of reasons for missed care and missed care in the OR. Nurse managers in the OR are critical in promoting nurses' job satisfaction, thus minimising the potential for missed care. To improve job satisfaction among OR nurses, nurse managers should invest in team-building exercises, effective communication training, and a culture that values collaboration, mutual respect, and continuous improvement. Nurse managers are well positioned to promote collaboration and interdisciplinary communication within the team. Fostering effective teamwork and communication can help prevent missed nursing care that arises from miscommunication or lack of coordination among different healthcare professionals. Additionally, providing support systems for OR nurses, such as debriefing sessions after challenging cases, can help alleviate stress and foster a sense of teamwork and job satisfaction. Nurse managers must advocate for necessary resources, including adequate staffing levels, appropriate equipment and supplies, and access to technology or tools that facilitate safe care provision. Appropriate resourcing will help to address job satisfaction and address some reported reasons for missed nursing care. Importantly, nurse managers are responsible for appropriate staffing levels and workload distribution and should ensure that an adequate number of qualified OR nurses are available to provide care to patients. By monitoring patient acuity and staff skill mix, staffing levels can be adjusted accordingly. Thus, OR nurse managers can help prevent situations where nurses are overwhelmed with excessive workloads, which may also lead to missed nursing care. Furthermore, they can encourage OR nurses to communicate their concerns, challenges, and workload issues.

## 5. Conclusions

This study showed that MNC in the OR setting is complex and is explained by several factors. Considering the modest effects found between the variables and the low variance explained by the final model, further studies are needed to identify other factors that may contribute to missed care in the OR. Nonetheless, our results are novel as we have identified relationships between the reasons for missed nursing care, competence, and frequency of missed nursing care, not previously explored. These relationships have not previously been identified, adding new understandings to this complex issue. Though further research is needed to confirm these findings, we present some key recommendations nurse managers can consider implementing to support their staff, reduce the missed care nurses are reporting in the OR, and improve patient outcomes.

## Figures and Tables

**Table 1:** Sample characteristics.

Characteristics	Sample	*n*	%
Participant variables			
Age in years (M, SD), range	535	46.02 (11.39), 21–75	
Sex	539		
Female		497	82.6
Male		42	7.0
Primary role	538		
Circulating/instrument nurse		304	50.5
Postanaesthetic recovery unit		43	7.1
Anaesthetic nurse		91	15.1
First surgical assistant (RN)		7	1.2
Multiple roles		17	2.8
Management		49	8.1
Educator		27	4.5
Qualifications	533		
Undergraduate education		412	77.3
Postgraduate education		121	22.7
OR nurse years of experience (M, SD), range	531	18.59 (11.64), 1–57	
<10 years		145	24.1
>10 years		386	64.1
Number of hours worked per week (M, SD), range	520	32.14 (11.78), 0–80	
Currently working full-time load (≥35 hrs/wk)		212	35.2
Number of overtime hours worked in the last 3 months (M, SD) range	525	26.40 (33.28), 0–240	
Number of shifts missed due to illness, injury, etc. in the last 3 months (M, SD) range	522	2.81 (5.17), 0–40	
Intention to leave	527		
In the next 6 months		72	12.0
In the next year		98	16.3
No plans to leave		357	59.3
Satisfaction (M, SD) range	547	10.65 (2.83), 3–15	
Hospital characteristics			
Number of ORs	533	9.75 (7.08), 1–40	
<6		224	37.2
>6		309	51.3
Surgery type	540		
Outpatient surgery		382	63.5
Inpatient surgery		472	78.4
Adult surgery		490	81.4
Paediatric surgery		360	59.8
Location (state/territory)	528		
Australian Capital Territory		10	1.7
Queensland		122	20.3
Northern Territory		4	0.7
New South Wales		132	21.9
Victoria		136	22.6
South Australia		51	8.5
Western Australia		35	5.8
Tasmania		19	3.2
International		19	3.2
Surgical speciality	540		
General surgery		466	77.4
Gynaecology		391	65.0
Urology		379	63.0
Orthopaedics		394	65.4
Transplant		60	10.0
Trauma		207	34.4
Burns		41	6.8
Plastics		336	55.8
Ears nose and throat		320	53.2
Dental/oral		296	49.2
Neurosurgery		139	23.1
Ophthalmology		219	36.4
Vascular		217	36.0
Cardiac		100	16.6
Thoracic		124	20.6

*Note.* M = mean; SD = standard deviation; OR = operating room; RN = registered nurse.

**Table 2:** Correlation matrix of study variables.

	Variables	1	2	3	4	5	6	7	8	9	10	11	12
1	Frequency of missed care in OR^a^	—											
2	Reasons for missed care in OR^b^	0.372^*∗∗*^	—										
3	Perceived perioperative competence^c^	−0.207^*∗∗*^	−0.059	—									
4	Sex	0.068	−0.041	−0.147^*∗∗*^	—								
5	Age (years)	−0.200^*∗∗*^	−0.201^*∗∗*^	0.305^*∗∗*^	−0.140^*∗∗*^	—							
6	Number of ORs in hospital	0.044	0.104^*∗*^	−0.074	−0.042	−0.048	—						
7	Postgraduate education	0.034	0.04	0.067	0.068	−0.042	0.079	—					
8	Hours per week worked	0.039	−0.013	0.083	0.101^*∗*^	−0.131^*∗∗*^	0.098^*∗*^	0.067	—				
9	Years of exp in OR	−0.082	−0.076	0.394^*∗∗*^	−0.156^*∗∗*^	0.782^*∗∗*^	0.01	−0.062	−0.142^*∗∗*^	—			
10	Intention to leave	0.096^*∗*^	0.111^*∗*^	0.023	0.00	0.043	0.031	−0.028	−0.058	0.079	—		
11	Overtime in last 3 months	0.053	−0.043	0.127^*∗∗*^	0.044	−0.065	−0.072	0.007	0.340^*∗∗*^	−0.048	−0.028	—	
12	Missed shifts in last 3 months	0.090	0.107^*∗*^	0.002	0.003	−0.179^*∗∗*^	0.06	−0.069	0.032	−0.138^*∗∗*^	0.031	0.052	—
13	Nurse job satisfaction^d^	−0.306^*∗∗*^	−0.299^*∗∗*^	0.08	−0.004	0.111^*∗*^	−0.027	0.122^*∗∗*^	0.051	0.00	−0.302^*∗∗*^	−0.044	−0.125^*∗∗*^

OR = operating room. *Note.*^*∗*^Spearman's correlation is significant at the 0.05 level (2-tailed); ^*∗∗*^significant at the 0.01 level (2-tailed). a = MISSCARE survey-operating room, b = perceived perioperative competence scale-short form (PPCS-SF), c = MISSCARE survey-operating room and d = job satisfaction scale.

**Table 3:** Parameter estimates for the model predicting frequency of missed care in the operating room.

Paths	*B*	SE *B*	*p*	*β*
*Direct paths*				
Age ⟶ frequency of missed care	−0.007^*∗*^	0.003	0.021	−0.166
Years of experience ⟶ frequency of missed care	0.005	0.003	0.071	0.131
Intention to leave ⟶ frequency of missed care	−0.031	0.045	0.492	−0.032
Satisfaction ⟶ frequency of missed care	−0.094^*∗∗*^	0.027	0.001	−0.203
PPC ⟶ frequency of missed care	−0.169^*∗∗∗*^	0.046	<0.001	−0.183
RMC ⟶ Frequency of missed care	0.224^*∗∗∗*^	0.041	<0.001	0.300
Age ⟶ PPC	0.002	0.003	0.512	0.046
Years of experience ⟶ PPC	0.015^*∗∗∗*^	0.003	<0.001	0.351
Age ⟶ RMC	−0.015^*∗∗∗*^	0.004	<0.001	−0.282
PPC ⟶ RMC	0.002	0.036	0.956	0.002
Years of experience ⟶ RMC	0.007	0.004	0.076	0.128
Intention to leave ⟶ RMC	−0.031	0.063	0.623	−0.024
Satisfaction ⟶ RMC	−0.220^*∗∗∗*^	0.037	<0.001	−0.357
*Covariances*				
Age ↔ years of experience	105.37^*∗∗∗*^	7.277	<0.001	0.797
Intention to leave ↔ satisfaction	−0.18^*∗∗∗*^	0.024	<0.001	−0.396
*Indirect effects*				
Age ⟶ RMC ⟶ frequency of missed care	−0.004^*∗∗∗*^	0.001	<0.001	−0.108
Years of experience ⟶ PPC ⟶ frequency of missed care	−0.003^*∗∗*^	0.001	0.002	−0.069
Satisfaction ⟶ RMC ⟶ frequency of missed care	−0.054^*∗∗∗*^	0.012	<0.001	−0.117
*Total effects*				
Age ⟶ frequency of missed care	−0.009^*∗∗∗*^	0.002	<0.001	−0.227
Years of experience ⟶ frequency of missed care	−0.004^*∗*^	0.002	0.043	−0.098
Satisfaction ⟶ frequency of missed care	−0.146^*∗∗∗*^	0.026	<0.001	−0.317

PPC = perceived perioperative competence; RMC = reasons for missed care. *Note*. ^*∗*^*p* ≤ 0.050; ^*∗∗*^*p*=0.01 level; ^*∗∗∗*^*p*=0.001 level.

## Data Availability

The data are not publicly available due to ethical approval requirements.

## References

[1] Kalisch B. J., Landstrom G. L., Hinshaw A. S. (2009). Missed nursing care: a concept analysis. *Journal of Advanced Nursing*.

[2] Ball J. E., Griffiths P., Rafferty A. M., Lindqvist R., Murrells T., Tishelman C. (2016). A cross-sectional study of ‘care left undone’ on nursing shifts in hospitals. *Journal of Advanced Nursing*.

[3] Jones T. L., Hamilton P., Murry N. (2015). Unfinished nursing care, missed care, and implicitly rationed care: state of the science review. *International Journal of Nursing Studies*.

[4] Recio-Saucedo A., Dall’Ora C., Maruotti A. (2018). What impact does nursing care left undone have on patient outcomes? Review of the literature. *Journal of Clinical Nursing*.

[5] Tubbs-Cooley H. L., Pickler R. H., Younger J. B., Mark B. A. (2015). A descriptive study of nurse-reported missed care in neonatal intensive care units. *Journal of Advanced Nursing*.

[6] Griffiths P., Recio-Saucedo A., Dall’Ora C. (2018). The association between nurse staffing and omissions in nursing care: a systematic review. *Journal of Advanced Nursing*.

[7] Chaboyer W., Harbeck E., Lee B.-O., Grealish L. (2021). Missed nursing care: an overview of reviews. *The Kaohsiung Journal of Medical Sciences*.

[8] Aiken L. H., Clarke S. P., Sloane D. M. (2001). Nurses’ reports on hospital care in five countries. *Health Affairs*.

[9] Aiken L. H., Sloane D. M., Bruyneel L. (2014). Nurse staffing and education and hospital mortality in nine European countries: a retrospective observational study. *The Lancet*.

[10] Clarke S. P., Aiken L. H. (2003). Failure to Rescue: needless deaths are prime examples of the need for more nurses at the bedside. *AJN, American Journal of Nursing*.

[11] Kalisch B. J., Tschannen D., Lee H., Friese C. R. (2011a). Hospital variation in missed nursing care. *American Journal of Medical Quality*.

[12] Kalankova D., Žiaková K., Kurucova R. (2019). Approaches to understanding the phenomenon of missed/rationed/unfinished care-a literature review. *Central European Journal of Nursing and Midwifery*.

[13] Mandal L., Seethalakshmi A., Rajendrababu A. (2020). Rationing of nursing care, a deviation from holistic nursing: a systematic review. *Nursing Philosophy: An International Journal for Healthcare Professionals*.

[14] Vincelette C., Thivierge-Southidara M., Rochefort C. M. (2019). Conceptual and methodological challenges of studies examining the determinants and outcomes of omitted nursing care: a narrative review of the literature. *International Journal of Nursing Studies*.

[15] Marsh V., Kalisch B., McLaughlin M., Nguyen L. (2020). Nurses’ perceptions of the extent and type of missed perioperative nursing care. *Association of periOperative Registered Nurses Journal*.

[16] Uçak A., Cebeci F. (2021). Competency in operating room nursing: a scoping review. *Journal of Education and Research in Nursing*.

[17] Gillespie B. M., Chaboyer W., Wallis M., Chang H.-Y. A., Werder H. (2009). Operating theatre nurses’ perceptions of competence: a focus group study. *Journal of Advanced Nursing*.

[18] Gillespie B., Hamlin L. (2009). A synthesis of the literature on “competence” as it applies to perioperative nursing. *Association of periOperative Registered Nurses Journal*.

[19] Gillespie B., Polit D. F., Hamlin L., Chaboyer W. (2012). Developing a model of competence in the operating theatre: psychometric validation of the perceived perioperative competence scale-revised. *International Journal of Nursing Studies*.

[20] Fukada M. (2018). Nursing competency: definition, structure and development. *Yonago Acta Medica*.

[21] Riley R., Peters G. (2000). The current scope and future direction of perioperative nursing practice in Victoria, Australia. *Journal of Advanced Nursing*.

[22] Bragadóttir H., Burmeister E. A., Terzioglu F., Kalisch B. J. (2020). The association of missed nursing care and determinants of satisfaction with current position for direct-care nurses—an international study. *Journal of Nursing Management*.

[23] Burmeister E. A., Kalisch B. J., Xie B. (2019). Determinants of nurse absenteeism and intent to leave: an international study. *Journal of Nursing Management*.

[24] Kalisch B. J., Tschannen D., Lee K. H. (2011b). Do staffing levels predict missed nursing care?. *International Journal for Quality in Health Care*.

[25] von Elm E., Altman D. G., Egger M., Pocock S. J., Gøtzsche P. C., Vandenbroucke J. P. (2014). The strengthening the reporting of observational studies in Epidemiology (STROBE) statement: guidelines for reporting observational studies. *International Journal of Surgery*.

[26] Kalisch B. J., Williams R. A. (2009). Development and psychometric testing of a tool to measure missed nursing care. *The Journal of Nursing Administration*.

[27] Kalisch B., McLaughlin M., Marsh V., Nguyen L., Talsma A. (2021). The development and testing of the MISSCARE survey OR. *Journal of Nursing Measurement*.

[28] Gillespie B. M., Harbeck E., Sutherland-Fraser S., Nicholson P., Boric T. (2023). Psychometric validation of the perceived perioperative competence scale-revised short form. *Journal of Advanced Nursing*.

[29] Harris P. A., Taylor R., Minor B. L. (2019). The REDCap consortium: building an international community of software platform partners. *Journal of Biomedical Informatics*.

[30] Harris P. A., Taylor R., Thielke R., Payne J., Gonzalez N., Conde J. G. (2009). Research electronic data capture (REDCap)—a metadata-driven methodology and workflow process for providing translational research informatics support. *Journal of Biomedical Informatics*.

[31] Polit D., Beck C. (2020). *Essentials of Nursing Research: Appraising Evidence for Nursing Practice*.

[32] Polit D. F., Yang F. (2016). *Measurement And the Measurement of Change: A Primer for the Health Professions*.

[33] Hu L. T., Bentler P. M. (1999). Cutoff criteria for fit indexes in covariance structure analysis: conventional criteria versus new alternatives. *Structural Equation Modeling: A Multidisciplinary Journal*.

[34] Nijkamp N., Foran P. (2021). The effects of staffing practices on safety and quality of perioperative nursing care-an integrative review. *Journal of Perioperative Nursing*.

[35] Sykes M., Gillespie B. M., Chaboyer W., Kang E. (2015). Surgical team mapping: implications for staff allocation and coordination. *Association of periOperative Registered Nurses Journal*.

[36] Müller P., Tschan F., Keller S. (2018). Assessing perceptions of teamwork quality among perioperative team members. *Association of periOperative Registered Nurses Journal*.

[37] Tørring B., Gittell J. H., Laursen M., Rasmussen B. S., Sørensen E. E. (2019). Communication and relationship dynamics in surgical teams in the operating room: an ethnographic study. *BMC Health Services Research*.

[38] Gillespie B., Steel C., Kang E. (2017). Evaluation of a brief team training intervention in surgery: a mixed-methods study. *Association of periOperative Registered Nurses Journal*.

[39] Gillespie B. M., Withers T. K., Lavin J., Gardiner T., Marshall A. P. (2016). Factors that drive team participation in surgical safety checks: a prospective study. *Patient Safety in Surgery*.

[40] Blackman I., Lye C. Y., Darmawan I. G. N. (2018). Modeling missed care: implications for evidence-based practice. *Worldviews on Evidence-Based Nursing*.

[41] Kalisch B. J., Xie B. (2014). Errors of omission: missed nursing care. *Western Journal of Nursing Research*.

